# Supplementary material for: Spatial Restrictions in Chemotaxis Signaling Arrays: A Role for Chemoreceptor Flexible Hinges across Bacterial Diversity
Source: Int J Mol Sci. 2019 Jun 19;20(12):2989. doi: 10.3390/ijms20122989 (PMC6628036; doi:10.3390/ijms20122989)
Supplement: Supplementary file 1 [file ijms-20-02989-s001.pdf]

International Journal of *Molecular Sciences*
MDPI

Supplementary material

Supplementary figures:

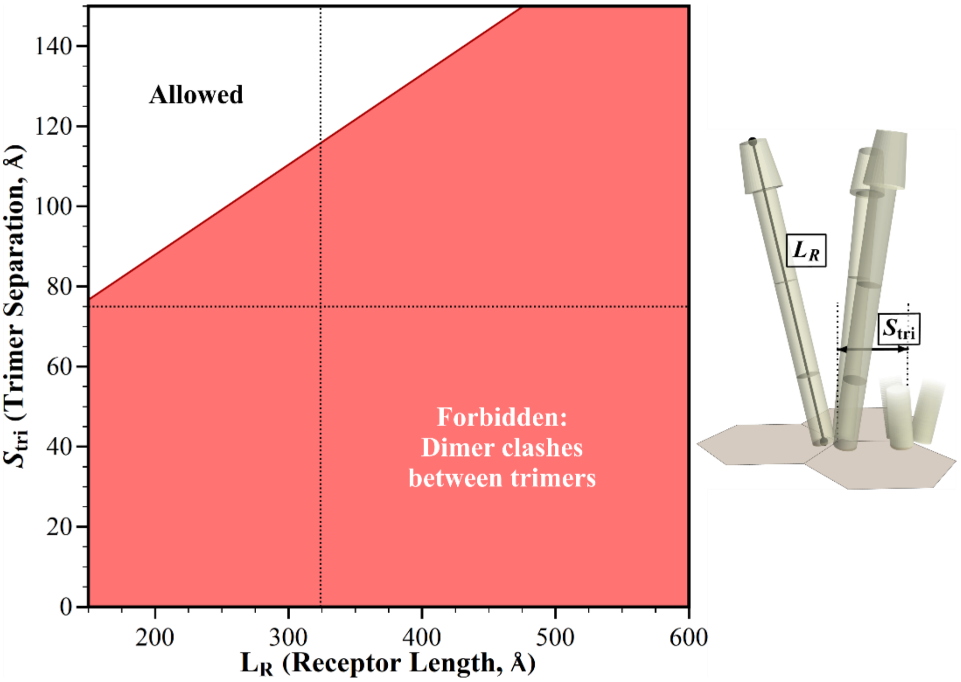

Figure S1. Geometric restrictions in core signaling complexes and their arrays as a function of trimer separation, S_{tri} and receptor length, L_R. The plot shows the structural consequences of combinations of trimer separation and receptor length. For allowed combinations (white area) receptors fit into the available space. For forbidden combinations they do not because of structural clashes between dimers in neighboring trimers (red areas). Dashed lines show the postulated shortest and longest chemoreceptor length as in Fig. 3. Dotted lines show respective values for *E. coli* receptors.

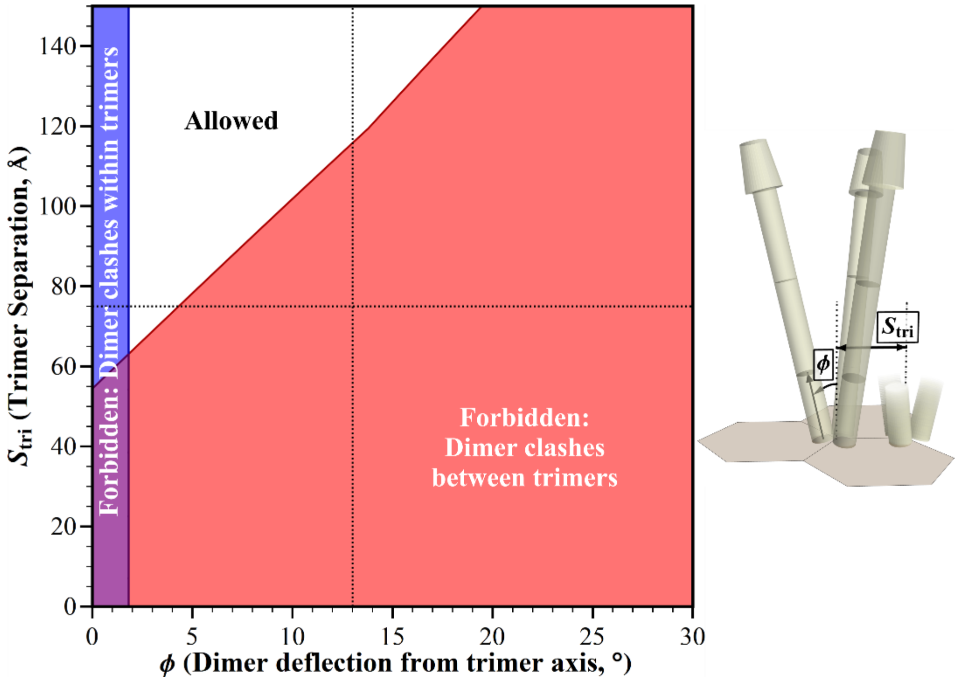

Figure S2. Geometric restrictions in core signaling complexes and their arrays as a function of trimer separation, S_{tri} and dimer deflection, ϕ. The plot shows the structural consequences of combinations of trimer separation and dimer deflection from the trimer central axis. For allowed combinations (white area) receptors fit into the available space. For forbidden combinations they do not because of structural clashes between dimers in neighboring trimers (red areas) or in the same trimer (blue areas). Dotted lines show respective values for *E. coli* receptors.

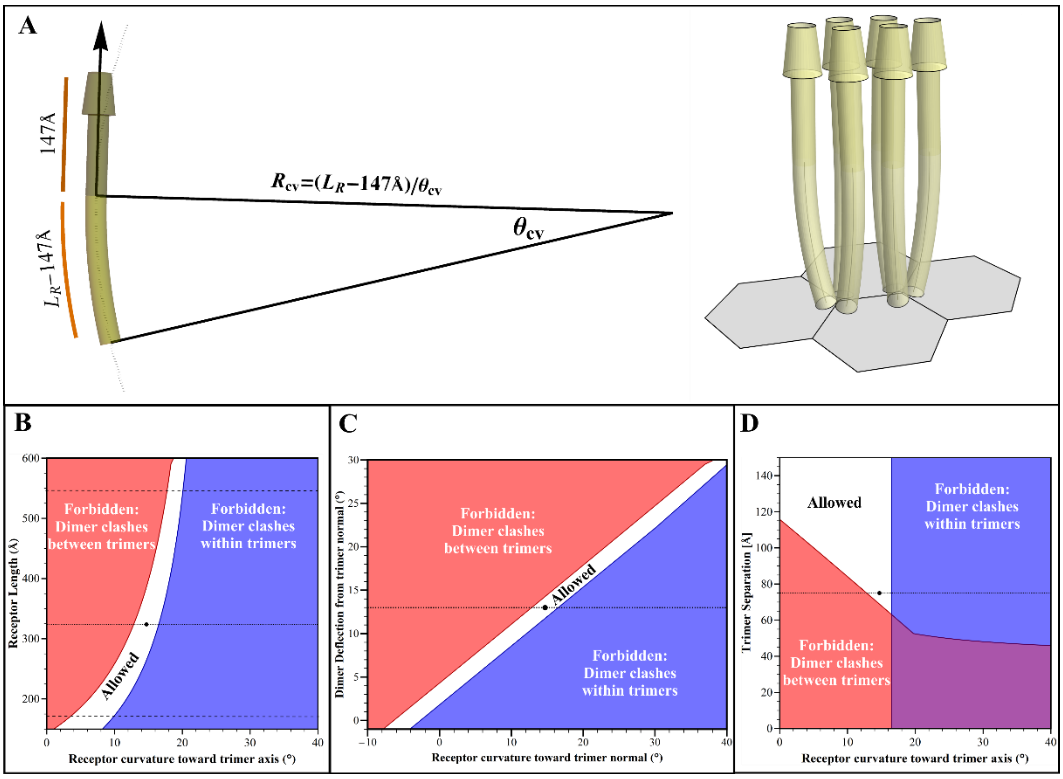

Figure S3. Geometric restrictions in core signaling complexes and their arrays as a function of curvature of the chemoreceptor coiled coil and other geometric parameters. A. Curvature of the coiled-coil segment was defined by the angle of the arc tracing the length of the central axis of that segment. This is illustrated for a single dimer in the left-hand diagram for an angle of 15° and for that same curvature of the dimers in a core complex in the right-hand diagram. B – D. The plots show structural consequences of combinations of curvature of the chemoreceptor coiled-coil segment with receptor length (B), dimer deflection (C) or trimer separation (D). For allowed combinations (white area) receptors fit into the available space. For forbidden combinations, they do not because of structural clashes between dimers in neighboring trimers (red areas) or in the same trimer (blue areas). Dashed lines show the postulated shortest and longest chemoreceptor length as in Fig. 3. Dotted lines show respective values for *E. coli* receptors and the larger dots on those lines mark the 15° curvature illustrated in panel A.

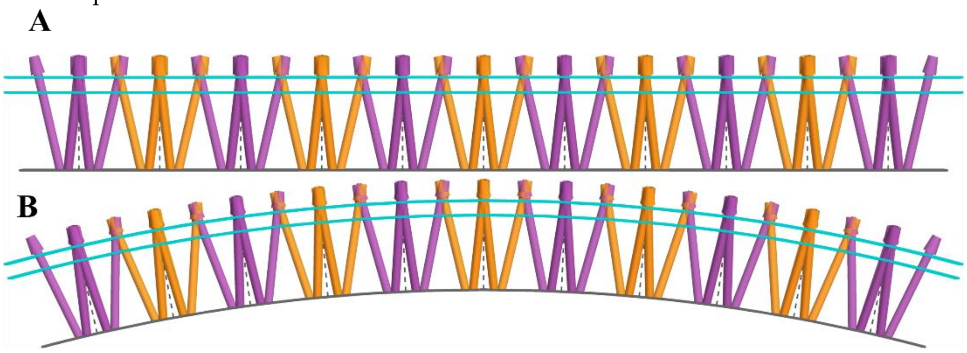

Figure S4. Minimal effect of polar membrane curvature on geometric restrictions in arrays of signaling complexes. Cross-sections of *E. coli* arrays are shown for straight receptors with the

position of the cytoplasmic membrane marked by lines representing its cytoplasmic and periplasmic surfaces. A. For a membrane lacking curvature, each trimer of dimers is oriented with its central axis (dashed arrow) normal to the membrane plane. In this situation, straight receptors clash. B. For a membrane with curvature corresponding to the 0.5 µm radius of an *E. coli* cell pole, each core complex is oriented with its central axis on a radius and thus the central axis of each trimer remains normal to the membrane. In this orientation, neighboring straight receptors in adjoining trimers are separated by ≈ 5 Å more than in the absence of membrane curvature. This additional separation is insufficient to avoid structural clashes.

Supplementary methods:

Modeling details

Chemoreceptor dimers were represented computationally as three *Cylinder* objects with a common diameter scaled to 25 Å plus a *Tube* object for which the top and bottom diameters could be different (Akkaladevi et al., 2018). Those objects represented, respectively, the receptor segment from the hexagonal baseplate to the Gly hinge (green in Fig. S5), the Gly hinge to the HAMP hinge (red in Fig. S5), the HAMP hinge to the membrane-proximal boundary of the frustum-shaped portion of the periplasmic, ligand-binding domain (blue in Fig. S5), and the frustum-shaped portion of that domain (gold in Fig. S5).

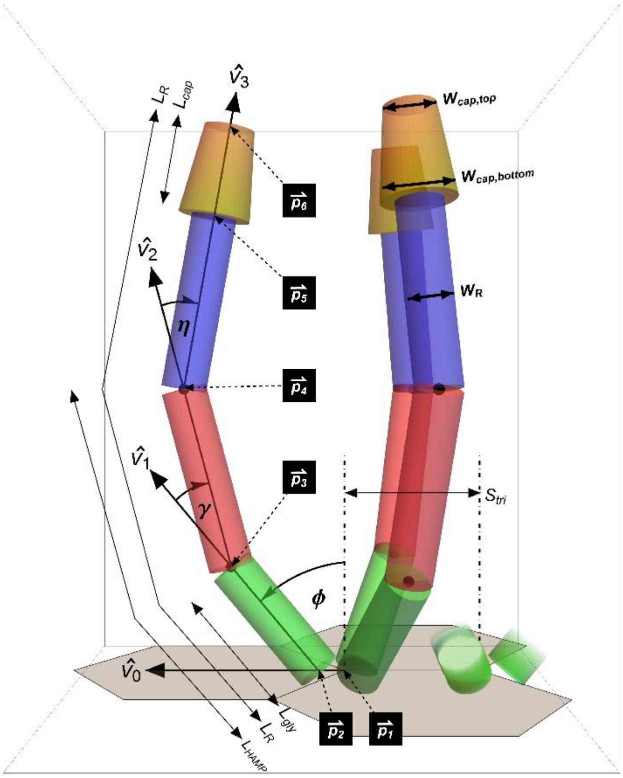

Figure S5. Dimer specifications. Each dimer in a trimer of dimers was represented by six coordinate points ($\overrightarrow{p_{1-6}}$), defined recursively, and the indicated lengths (L), widths (W) and vectors. The three constituent dimers in a trimer were anchored to the baseplate ($\overrightarrow{p_2}$), radially distributed around the central trimer axis, at each vertex of the hexagonal array ($\overrightarrow{p_1}$),. Trimers were separated by distance S_{tri} between their respective central axes. Dimers were deflected away from those axes with positive angle ϕ. Hinge deflections at $\overrightarrow{p_3}$ and $\overrightarrow{p_4}$ toward the central axis were defined as positive. See text for additional descriptions.

In the analyses for this study, hinge deflections were considered only at the Gly hinge. However, the HAMP hinge was included in the model for later use. The four objects were defined computationally by the coordinates of endpoints and associated widths (Fig. S5). Thus, a chemoreceptor dimer was defined by six characteristic points in three-dimensional space: 1) the

center of the trimer ($\overrightarrow{p_1}$), 2) the base of the dimer on the hexagonal base plate ($\overrightarrow{p_2}$), 3) the position of the glycine hinge ($\overrightarrow{p_3}$), 4) the position of the HAMP hinge ($\overrightarrow{p_4}$), 5) the position of the membrane-proximal base of the frustum cap ($\overrightarrow{p_5}$), and 6) the position of the membrane-distal tip of the cap ($\overrightarrow{p_6}$) (Fig. S5).

Three-dimensional trimers of dimers were created by placing three capped rods in contact at the membrane-distal tips of their cytoplasmic domains, translated $W_d/\sqrt{3}$ in the x-y plane, where W_d is the width of the dimer, centered on a hexagonal vertex and displaced from the central axis of that vertex by a designated angle. The direction of each dimer was represented by the in-plane unit vector $\widehat{v_0}$:

$$\overrightarrow{p_2} = \overrightarrow{p_1} + \frac{W_d}{\sqrt{3}}\widehat{v_0}$$

The constituent points of each dimer ($\overrightarrow{p_{2-6}}$) were defined by recurrence relations, with each successive coordinate a function of the previous. This provided a clean notation while ensuring that the receptor segments remained linked. The base segment of each dimer subtended an angle ϕ relative to the central axis of the trimer away from $+\hat{z}$ in the direction $\overrightarrow{v_0}$.

Coordinates of the glycine hinge were:

$$\overrightarrow{p_3} = \overrightarrow{p_2} + L_1\widehat{v_1} = \overrightarrow{p_2} + L_1 \sin(\phi)\,\widehat{v_0} + L_1 \cos(\phi)\,\hat{z}$$

with L_1 the scalar distance of the glycine hinge from the membrane-distal tip and $\widehat{v_1}$ the unit direction of the dimer section projecting from its base-plate-affixed tip in 3-D space, which is $\widehat{v_0}$ in the x-y direction and $\hat{z}$ in the z direction. Coordinates of the HAMP hinge were:

$$\overrightarrow{p_4} = \overrightarrow{p_3} + L_2\widehat{v_2} = \overrightarrow{p_3} + L_2 \sin(\phi-\gamma)\,\widehat{v_0} + L_2 \cos(\phi-\gamma)\,\hat{z}$$

with L_2 is the scalar distance separating the two hinges along the dimer long axis. In this formulation, a hinge deflection γ is positive towards the trimer center $-\widehat{v_0}$. Similarly, the membrane-distal and membrane proximal positions of the frustum were:

$$\overrightarrow{p_5} = \overrightarrow{p_4} + L_3\widehat{v_3} = \overrightarrow{p_4} + L_3 \sin(\phi-\gamma-\eta)\,\widehat{v_0} + L_3 \cos(\phi-\gamma-\eta)\,\hat{z}$$

$$\overrightarrow{p_6} = \overrightarrow{p_5} + L_4\widehat{v_3} = \overrightarrow{p_5} + L_4 \sin(\phi-\gamma-\eta)\,\widehat{v_0} + L_4 \cos(\phi-\gamma-\eta)\,\hat{z}$$

with η deflection at the HAMP hinge, positive toward the trimer center. L_3 and L_4 were the scalar distances along the receptor body from the HAMP hinge to the membrane-proximal and membrane-distal boundaries of the conical frustum, respectively (Fig. S5).

Receptor clashes

Clashes can be between dimers in the same trimer (intra-trimer) or in neighboring trimers (inter-trimer). Inter-trimer clashes determine the minimum hinge deflection that avoids clashes. Intra-trimer clashes define the maximum deflection without clashes. As parameters are varied, intra- or inter-timer collisions each occur first at one of four edges: 1) the membrane-proximal edge of the cylinder between the base plate and the glycine hinge (marked with a green disc in Fig. S6), 2) the top of the cylinder between the two hinges (red disc, Fig. S6), 3) the membrane- proximal boundary of conical frustum (gold disc, Fig. S6) and 4) the membrane-distal boundary of that frustum (gold disc, Fig. S6).

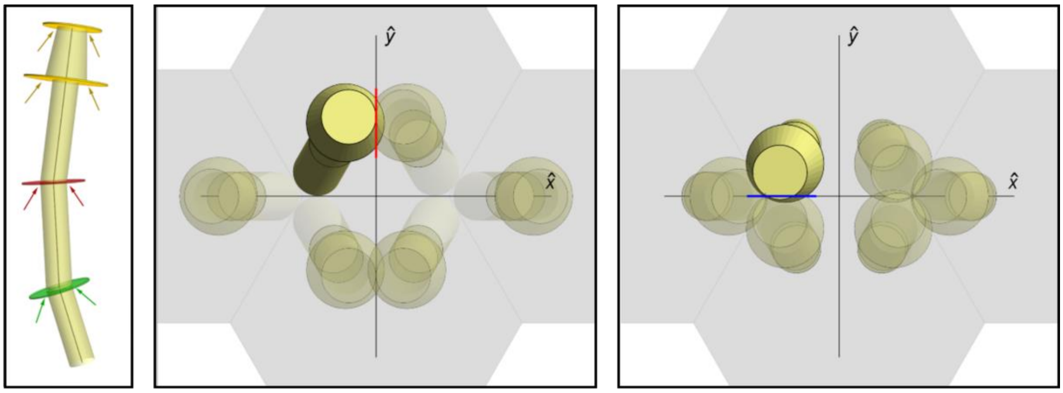

Figure S6. Dimer clashes. Left panel. The four edges at which clashes first occur among dimers within chemoreceptor arrays as parameters are varied are marked with discs and arrows (see text). Center and right-hand panels. A coordinate system to assess dimer clashes. Each panel shows two trimers of chemoreceptor dimers in a signaling array with the origin of the coordinate system midway between the trimers. One of the dimers undergoing clashing is highlighted. Clashes occur if dimers cross the y-axis in an inter-trimer collision (center panel) or the x-axis in an intra-trimer collision (right-hand panel). Modeled dimers are non-interacting, so clashing dimers occupy the same volume.

The points of first collision on these edges depend upon the relative orientation of the potentially colliding dimers. We defined those orientations using a coordinate system with its origin at the midpoint between the two trimers of a core complex. Thus trimer centers were thus given by $\overrightarrow{p_1} = \{\pm S_{tri}/2,0,0\}$, where S_{tri} is the distance between two neighboring trimers. The in-plane vectors for each trimer, described by $\widehat{v_0}[\theta] = \{\cos[\theta], \sin[\theta], 0\}$, are given by the angles 60°, 180°, and 300° for the left trimer, and 0°, 120°, and 240° for the right. Taking the 60° dimer as reference (top left in Fig. S6 middle), and with the condition that all dimers experience the same hinge deflections, symmetry dictates that inter-trimer collisions occur upon crossing the y-axis (x<0); likewise, intra-trimer collisions occur upon crossing the x-axis (y>0). This is an especially powerful assignment as it dramatically reduces the complexity of the analysis, requiring only a single component of a single dimer versus a consideration of the full 3-D space.

The locations of collision points around the edge varies with the orientation of the constituent segment; these may be defined via the incorporation of an additional vector term ($\vec{u}$ and $\vec{w}$ for the inter- and intra- trimer collisions, respectively) to the characteristic vertex points $\overrightarrow{p_{3-6}}$:

$$\hat{u}_n[\hat{v}_n] = \left\{-\left(1-v_{n,x}^2\right)^{0.5}, \frac{v_{n,x}v_{n,y}}{\left(1-v_{n,x}^2\right)^{0.5}}, \frac{v_{n,x}v_{n,z}}{\left(1-v_{n,x}^2\right)^{0.5}}\right\}$$

$$\widehat{w}_n[\hat{v}_n] = \left\{\frac{-v_{n,x}v_{n,y}}{\left(1-v_{n,y}^2\right)^{.5}}, \left(1-v_{n,y}^2\right)^{0.5}, \frac{-v_{n,y}v_{n,z}}{\left(1-v_{n,y}^2\right)^{0.5}}\right\}$$

These unit vectors are dependent on and perpendicular to the unit direction $\hat{v}_n$ of the associated dimer section. Adding this vector, using the radius of the given segment, generates the explicit coordinate of each collision point. However, because of the origin definition we require only the x-component of the inter-trimer collision point and the y-component of the intra-trimer point to identify collisions:

$$\overrightarrow{p_{n,x}} + \frac{W_n}{2}\widehat{u_{n,x}}[\hat{v}_n] = \overrightarrow{p_{n,x}} - \frac{W_n}{2}\left(1-v_{n,x}^2\right)^{0.5} > 0$$

$$\overrightarrow{p_{n,y}} + \frac{W_n}{2}\widehat{w_{n,y}}[\hat{v}_n] = \overrightarrow{p_{n,y}} - \frac{W_n}{2}\left(1-v_{n,y}^2\right)^{0.5} < 0$$

Thus, clashes can be identified by considering eight points, four for intra-trimer and four for inter-trimer collisions.

Incorporating the previously defined coordinates and vectors yields generalized conditionals that are functions of user-defined geometries. There are 11 independent parameters, and the

allowable ranges of each can be explored by assigning values to those remaining and plotting the resulting inequalities:

- Trimer separation (S_{tri})
- Glycine hinge position ($L_{gly} = L_1$)
- HAMP hinge position ($L_{hamp} = L_2$)
- Cap height ($L_{cap} = L_4$)
- Total receptor length ($L_R = L_1 + L_2 + L_3 + L_4$)
- Receptor width (W_R)
- Receptor width ($W_{cap,B}$)
- Receptor width ($W_{cap,T}$)
- Deflection from the trimer axis (ϕ)
- Glycine hinge deflection toward the trimer axis (γ)
- HAMP hinge deflection toward the trimer axis (η)

Each coordinate $\overrightarrow{p_{1-6}}$ is defined so as to make use of fundamental dimer measurements: for example, L_R denotes the total receptor length including periplasmic geometry, and the glycine and HAMP hinges are independently positioned along the dimer's length based on their respective distances above the baseplate.

Examination of the above definitions reveal a number of inherent restrictions. In the absence of the conical frustrum top, parallel dimers (i.e. $\phi = 0°$) would be allowed; however, the additional widths ascribed to the frustum necessarily generates a minimum required value for deflection from the trimer axis. The glycine hinge (γ), acting at $\overrightarrow{p_3}$, can only affect the three collisions above this point ($\overrightarrow{p_{4,5,6}}$); likewise, the HAMP hinge (η) is limited to affecting collisions at points $\overrightarrow{p_{5,6}}$. The trimer separation S_{tri} is independent of any dimer specifications, and thus has no bearing on intra-trimer collisions. Dimers within a trimer are placed as tangent diameters dependent on the receptor width.

In our analyses, we focused on the interplay between pairs of parameters. One-dimensional and three-dimensional analyses are also possible. Two-dimensional relationships were plotted using Mathematica's *RegionPlot*. While each of the eight possible collision points was defined separately (Fig. S7, left), inter- and intra-trimer collisions were grouped as single Boolean units to yield unified behavior for each (Fig. S7, right, insets), and combined to show the overall pattern of allowed and forbidden receptor orientations (Fig. S7, right).

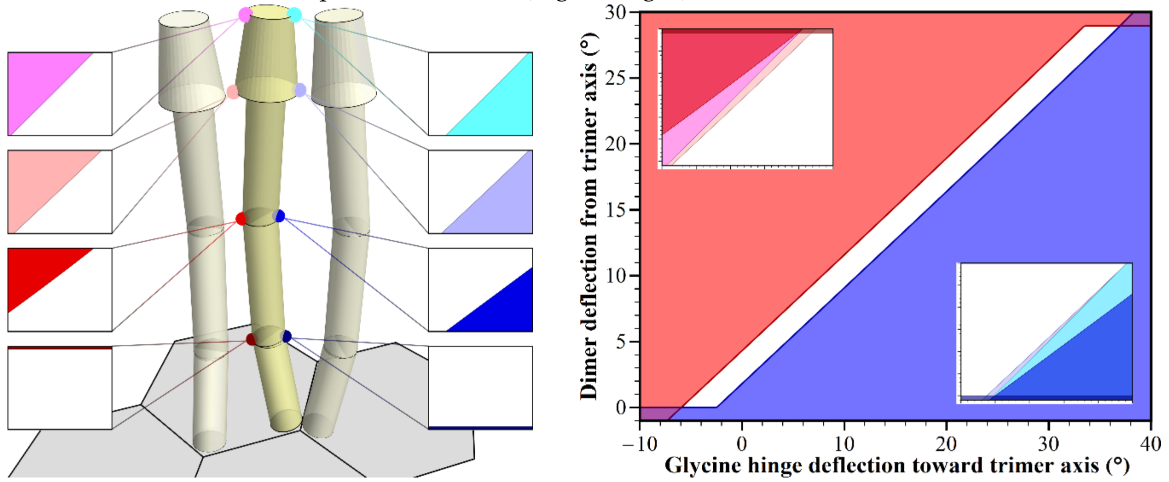

Figure S7. Plotting allowed and forbidden parameter combinations. Left panel. Collision points on one chemoreceptor dimer in an array are marked with colored balls. Plots of dimer collisions as a function of glycine hinge deflection (abscissa) and dimer deflection from the trimer axis (ordinante) are shown on the left for each inter-trimer collision point and on the right for each intra-trimer collision point, with the color of the forbidden region corresponding to the color of the respective ball. Note that for the Gly hinge collision points, the forbidden regions are narrow segments at the top and bottom of the lowest plots on the left and right, respectively. Scales on the two axes for the eight

small plots are the same as the larger plot on the right. Right panel. The upper inset shows the combined plots for the four inter-trimer collision points and the lower inset the combined plots for intra-trimer collision points. The larger plot combines forbidden and allowed regions for all eight collision points with inter-trimer collisions in red and intra-trimer collisions in blue.

Interactive Modeling Utility

Our modeling tool is available as a Mathematica Computable Document Format (cdf) file that can be downloaded from https://doi.org/10.32469/10355/68127 . It requires installation of the free Wolfram Player [https://www.wolfram.com/player/] or the full Mathematica suite. The free CDF player restricts file import/export, so models and plots are saved as screenshots. The utility generates interactive models and plots based on user-defined parameters (Fig S8), starting with default values for *E. coli* (Akkaladevi et al., 2018). As parameters are changed using sliders, the utility displays the resulting geometry of a single core complex. Optional graphics controls adjust the angle from which the model is viewed. Two-dimensional plots showing forbidden and allowed combinations of two, operator-chosen variable parameters are generated from operator-chosen values for fixed parameters and the range of variable parameters. A 'plot marker' option places a point on the plot corresponding to the current values, and will adjust as those values are altered. This allows users to correlate the plot to the physical geometry in real space.

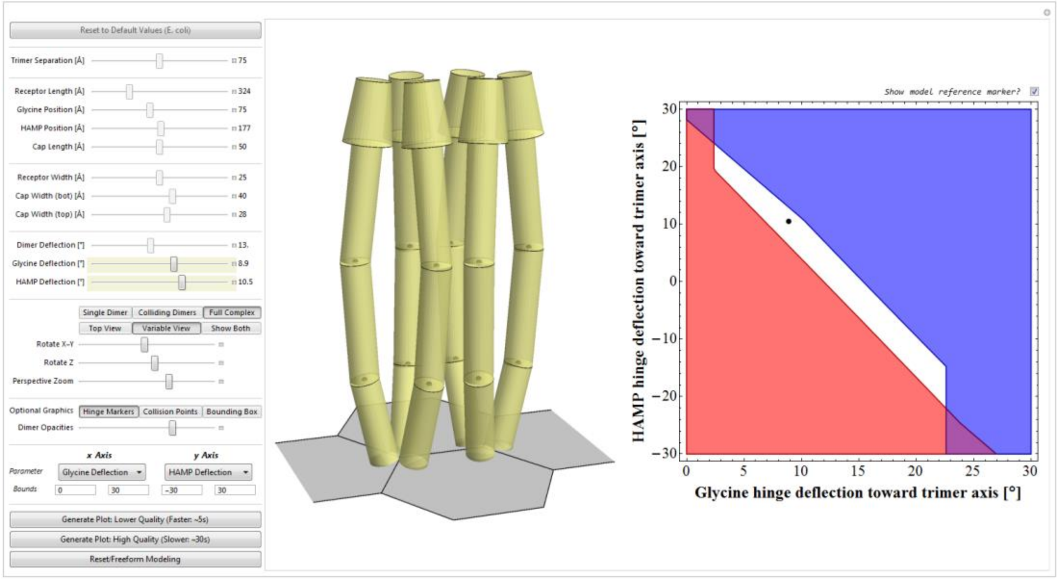

Figure S8. Screenshot of the downloadable modeling/plotting utility.
